# Transfusion training for haematology registrars: Results of a UK‐wide survey

**DOI:** 10.1111/tme.13146

**Published:** 2025-05-19

**Authors:** Lorna Cain, Lucy Neave, Shubha Allard, Dora Foukaneli, Suzy Morton, Shruthi Narayan, The Royal College of Pathologists Transfusion Medicine Specialty Advisory Committee

**Affiliations:** ^1^ Haematology/Transfusion Medicine, NHS Blood and Transplant Oxford UK; ^2^ Nuffield Division of Clinical Laboratory Sciences University of Oxford Oxford UK; ^3^ Department of Haematology Guys and St. Thomas's Hospital London UK; ^4^ Haematology/Transfusion Medicine NHS Blood and Transplant, Colindale London UK; ^5^ RCPath Transfusion Medicine Specialty Advisory Committee London UK; ^6^ Haematology/Transfusion Medicine, NHS Blood and Transplant Cambridge UK; ^7^ Department of Haematology Cambridge University Hospitals NHS Foundation Trust Cambridge UK; ^8^ Haematology/Transfusion Medicine, NHS Blood and Transplant Birmingham UK; ^9^ Department of Haematology University Hospitals Birmingham NHS Foundation Trust Birmingham UK; ^10^ Serious Hazards of Transfusion (SHOT), Manchester UK; ^11^ Donor Medicine, Haematology/Transfusion Medicine, NHS Blood and Transplant Manchester UK

**Keywords:** haematology training, post‐graduate education, survey, transfusion medicine

## Abstract

**Objectives:**

To understand the current status of transfusion training for haematology specialty registrars in the UK and identify potential solutions for improvement.

**Background:**

Transfusion knowledge and experience are essential for all haematologists. There are concerns regarding inconsistencies with the delivery of transfusion training.

**Methods:**

A 30‐question online survey was distributed using the SurveyMonkey platform to UK haematology specialty trainees in June–July 2023.

**Results:**

A total of 150 responses (response rate 24%) were received from trainees at different stages of training in 17 training regions. Forty‐four percent (66/150) trainees had undertaken or were expected to undertake a dedicated transfusion post during their training; these were deemed to be educationally useful. Ninety‐nine percent (149/150) trainees had managed transfusion queries at work. Most trainees (69%, 103/150) had received teaching in hospitals outside of a dedicated transfusion post. A high proportion (87%, 131/150) had attended a transfusion course provided by a national blood service.

Levels of overall satisfaction with the transfusion training provided varied: very satisfied/satisfied (30%), neutral (31%), dissatisfied/very dissatisfied (39%). The most common barriers to training selected were lack of exposure to the transfusion laboratory (75%), clashing clinical commitments taking priority (74%), and lack of provision of dedicated transfusion training (70%).

**Conclusion:**

There is mixed satisfaction with the transfusion training received by UK haematology registrars, evident from this survey. Protected time for transfusion training in dedicated transfusion posts and in other hospital posts, including laboratory time, is needed for all haematology trainees.

## 1 INTRODUCTION

Knowledge and experience of transfusion is essential for all haematologists; patients directly under the care of haematologists are high users of blood components, often with complex transfusion requirements, and transfusion queries are frequent in liaison work with other clinical specialities. Haemovigilance data have shown repeatedly that transfusion incidents are frequently attributable to a lack of knowledge, and therefore ensuring robust transfusion training for haematology trainees is essential for patient safety.^1^ The Infected Blood Inquiry (IBI) report, published in 2024, specifically recommends that all staff involved in blood transfusion receive adequate transfusion training and that this is integral to improving patient outcomes.^2^

In contrast to some other countries, haematology training in the UK is a 5‐year whole‐time equivalent programme, from specialty trainee year 3–7 (ST3‐7), following 2 years of foundation training (FY1‐2), and at least 2 years of adult internal medicine training (IMT) or paediatric specialty training (Paediatrics ST), or gaining equivalent required experience.^3^ Haematology trainees must gain proficiency in haemato‐oncology, coagulation, red cell disorders, immuno‐haematology, paediatrics, as well as transfusion. Maintaining the quality of haematology training has faced many challenges in recent years, including a growing curriculum, changes in working patterns necessitated by the 2016 junior doctor contract, staff shortages, the drift of clinical haematology away from its laboratory base, and the COVID‐19 pandemic.^4^, ^5^

The last national survey of transfusion training in the UK, which was undertaken on behalf of the Royal College of Pathologists (RCPath) in 2008, highlighted dissatisfaction with transfusion training by haematology trainees.^6^, ^7^ More recently, variability in transfusion training has been highlighted to the RCPath Transfusion Medicine Specialty Advisory Committee (SAC) from informal feedback from trainees and committee members, especially between regions that do and do not have a dedicated transfusion post (a rotation in the 5 year training period primarily focused on transfusion). Additional concerns have been raised owing to poor performance by candidates in the transfusion questions of the Part 1 and Part 2 FRCPath haematology examinations.^8^

This survey was undertaken by the RCPath Transfusion SAC to update the current picture of transfusion training across the UK for haematology specialty trainees from a trainee perspective, focusing on training opportunities, barriers to training, and potential solutions.

## 2 METHODS

### 2.1 Development of the survey tool

A 30‐question survey tool was designed with questions relating to: demographics, dedicated transfusion posts, hospital‐based training, transfusion teaching and courses, trainee overall assessment of transfusion training, barriers to training and potential solutions (Data S1). The final version of the survey tool was approved by the RCPath Transfusion SAC and RCPath Professionalism and Learning teams. The survey was additionally co‐badged by the British Society of Haematology (BSH) education committee and Joint Royal Colleges of Physicians Training Board (JRCPTB) Haematology SAC. The survey was hosted on the RCPath SurveyMonkey^9^ account, and the online survey was piloted by five haematology specialty trainees who did not identify any issues with usability.

### 2.2 Eligibility and survey distribution

All trainees enrolled in the JRCPTB haematology specialty training programme at the time of survey completion were eligible to participate. This included trainees of all grades, those currently out‐of‐programme and from all regions of the UK. Doctors working in staff grade posts who might apply for specialist registration by the Portfolio Pathway (formerly known as CESR) were excluded. The survey link was distributed by: emails to regional haematology registrar representatives who were asked to forward the survey link to all the trainees in their region; cascading via the HaemSTAR network^10^ regional representatives; and X.^11^ The survey was open from 12 June to 21 July 2023. Responses were anonymous. Participation was voluntary and consent was implied by survey completion. Trainees were informed that the results would be used to inform future transfusion training.

### 2.3 Data analysis

The survey form required completion of all questions for submission, resulting in no missing data. The results are presented using descriptive statistics. Free text survey data were reviewed by the co‐authors, with the selection of illustrative comments. The total number of haematology specialty trainees was obtained from the JRCPTB.

## 3 RESULTS

### 3.1 Responses received

Six hundred and twenty‐seven JRCPTB‐registered haematology specialty trainees were eligible to participate; 150 survey responses were received (response rate 24%). The respondents were from 17 of 20 training regions, with representation from ST3 to ST7 (Table 1).

**TABLE 1:** Characteristics of survey respondents.

Characteristic	*N* (%)
*Stage of training*	
ST3	13 (9)
ST4	34 (23)
ST5	33 (22)
ST6	44 (29)
ST7	26 (17)
*FRCPath Haematology examinations completed*	
None	44 (29.3)
Haematology Part 1 only	55 (36.7)
Haematology Part 1 and Part 2	51 (34.0)
*Deanery*	
East Midlands^a^	9 (6)
East of England	5 (3)
London^b^	43 (29)
Mersey	2 (1)
North East	8 (5)
North Western	7 (5)
Northern Ireland	9 (6)
Peninsula	6 (4)
Scotland—North	3 (2)
Scotland—South East	1 (1)
Scotland—West	5 (3)
Severn	5 (3)
Thames Valley	3 (2)
Wales	8 (5)
Wessex	4 (3)
West Midlands	22 (15)
Yorkshire and Humber^c^	10 (7)

*Note*: Total *N* = 150. Subdivisions of training region reported by trainees are given in the below footnotes.

^a^ East Midlands—North (*N* = 7).

^b^ London—East (*N* = 1), North Central (*N* = 20), North Central East (*N* = 1), North East (*N* = 1), North West (*N* = 7), South (*N* = 7), South East (*N* = 1).

^c^ Yorkshire and Humber—East/West (*N* = 4), South (*N* = 2).

### 3.2 Transfusion posts

Forty‐eight (32%) trainees had undertaken a dedicated transfusion post, with a further 18 (12%) expecting to undertake a transfusion post during their training, from 13 of the 17 represented training regions.

Transfusion posts were at a: blood service only (15/48, 31%); hospital only (16/48, 33%); or both a blood service and hospital (17/48, 35%). Post durations reported were: more than 3 months (14/48, 29%); 1–3 months duration (26/48, 54%); 1 week to 1 month (6/48, 12%); less than a week (2/48, 4%).

The majority of those who had undertaken a transfusion post either strongly agreed (16/48, 33%), or agreed (18/48, 38%), with the statement ‘My transfusion post was useful educationally’. The remainder neither agreed or disagreed (6/48, 13%), disagreed (4/48, 8%) or strongly disagreed (4/48, 8%).

### 3.3 Hospital‐based transfusion training

Almost all trainees (99%, 149/150) had handled clinical transfusion queries at work, both in hours and on call (55%, 82/150), whilst on call (30%, 45/150), or as part of routine clinical work (15%, 22/150). The most frequently reported queries managed were transfusion reactions (94%, 141/150), special transfusion requirements/special components (93%, 139/150) and major haemorrhage/emergency transfusion (87%, 131/150) (Data S2, Table S1). The least frequently reported were paediatric transfusion (27%, 40/150) and antenatal transfusion (33%, 49/150). Overall, most trainees (67%, 101/150) had helped investigate a transfusion reaction or event, with 80% (56/70) of senior trainees (ST6/7) having experience with this.

Less than half of the trainees (43%, 65/150) had attended either a hospital transfusion team (HTT) or hospital transfusion committee (HTC) meeting. This was only slightly higher among senior trainees (ST6/7), 63% (44/70). Regarding the educational benefit of attending these meetings, of those who had attended either a HTT or HTC meeting, 9% (6/65) strongly agreed, 69% (45/65) agreed, 17% (11/65) neither agreed nor disagreed, 3% (2/65) disagreed, and 2% (1/65) strongly disagreed that this had been a useful educational experience. Regarding the frequency of attendance, of those who had provided a frequency of attendance, 57% (34/60) had attended only once.

### 3.4 Transfusion teaching and courses

Most trainees (73%, 109/150) had received transfusion teaching at their regional training days, although a significant number (27%, 41/150) reported that their regional training days had not covered transfusion topics. 69% (103/150) trainees had received teaching in the hospitals where they had worked outside of a dedicated transfusion post. The frequency of these teaching sessions was highly variable, from ‘weekly’ to ‘once per year or less’. Of those who had received hospital teaching, 85% (88/103) reported laboratory aspects of transfusion had been taught. However, only 41% (42/103) had spent time in the hospital transfusion laboratory in these sessions.

A high proportion of trainees had attended a transfusion medicine course (87%, 131/150) or planned to attend such a course (9%, 14/150) (Data S2, Table S2). These courses were highly regarded by the trainees. Almost half of respondents (49%, 74/150) had used online transfusion resources or e‐modules (Data S2, Table S3).

### 3.5 Trainee assessment of transfusion training

Respondents were asked about their confidence in answering transfusion queries during working hours; 44% (66/150) trainees felt able to deal with these independently, 49% (74/150) did not feel able to handle the calls independently but had consultant advice available, 5% (7/150) did not feel equipped and reported a lack of senior support, and 2% (3/150) were unsure. It was a similar picture for confidence with handling on‐call/out of hours transfusion calls; 42% (63/150) reported being able to handle these fairly independently, 52% (78/150) did not feel able to handle the calls independently but had consultant advice available, 5% (7/150) did not feel equipped and reported a lack of senior support, and 1% (2/150) were unsure. Of the senior trainees (ST6/7), 70% (49/70) felt able to deal with transfusion queries independently in hours, and 67% (47/70) out of hours.

With regards to supporting a hospital transfusion laboratory as a consultant, 21% (31/150) thought they would be equipped for clinical and laboratory aspects, 27% (41/150) thought they would be equipped for clinical aspects only, 32% (48/150) did not think their training programme would equip them to undertake this role, and 20% (30/150) were unsure. More trainees (53%, 80/150) felt their training would equip them to provide safe clinical transfusion advice to colleagues in other specialties, although 16% (25/150) did not think they would be equipped for this, and 30% (45/150) were unsure.

**FIGURE 1:**
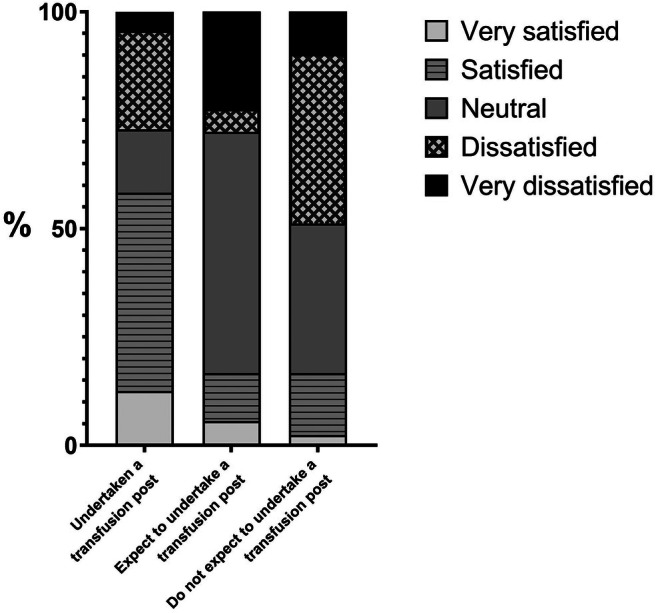
Overall satisfaction with transfusion training. Overall satisfaction with transfusion training grouped by completion of a transfusion post/expectation to undertake a transfusion post.

Overall satisfaction with transfusion training was mixed: very satisfied (6%, 9/150), satisfied (24%, 36/150), neutral (46%, 46/150), dissatisfied (30%, 45/150), very dissatisfied (9%, 14/150). 58% (28/48) of those who have completed a transfusion post were very satisfied/satisfied with their transfusion training compared to 17% (14/84) of those who do not expect to undertake such a post (Figure 1).

### 3.6 Barriers to training

**FIGURE 2:**
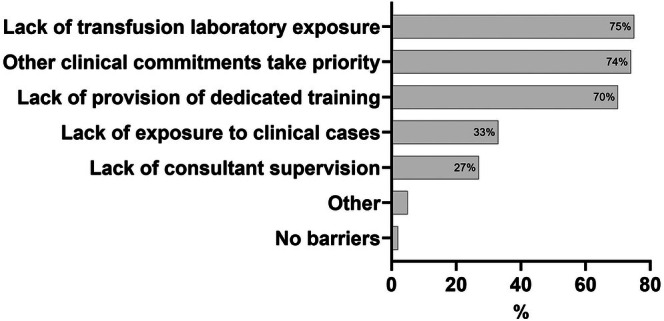
Barriers to transfusion training. Barriers to training as selected by respondents (*N* = 150). ‘Other’: Lack of transfusion consultant time (*n* = 3); difficulty accessing blood service transfusion courses due to funding (*n* = 3); lack of resources in the hospital trust (*n* = 1); transfusion service seems distant (*n* = 1).

The most common barrier to training selected was a lack of transfusion laboratory exposure, followed by other clinical commitments taking priority, and a lack of provision of dedicated training (Figure 2). Illustrative free text comments from four different trainees relating to the barriers to transfusion training are listed here.

Barriers free text comment 1:
  (Transfusion is) very much the inferior / forgotten 25% of haematology training – local to me. I think this is because: 1) the “culture” of going to the laboratory is absent across the whole department 2) “superspecialisation” means that those interested in transfusion are physically distant and others do not have an interest / or perhaps knowledge with which to teach or inspire.
  Barriers free text comment 2:
  In comparison to haem‐onc, general haem, and thrombosis & haemostasis, this topic receives less teaching/training time and also less clinical exposure – (it) mostly relates to on call management of acute scenarios such as major haemorrhage/transfusion reactions rather than wider training/exposure to the technical theory and lab aspects. (There is an) expectation that you will read around the topic and learn yourself, but (it is) hard to start doing this with little baseline knowledge.
  Barriers free text comment 3:
  Bare‐minimum staffing – leading to regular understaffing when colleagues are on‐call/on leave = no time for regular in‐hours teaching as clinical service provision takes priority over training.
  Barriers free text comment 4:
  (There is) No dedicated training. All learning happens as part of requested study leave or in my own time.

### 3.7 Solutions for improvement

**FIGURE 3:**
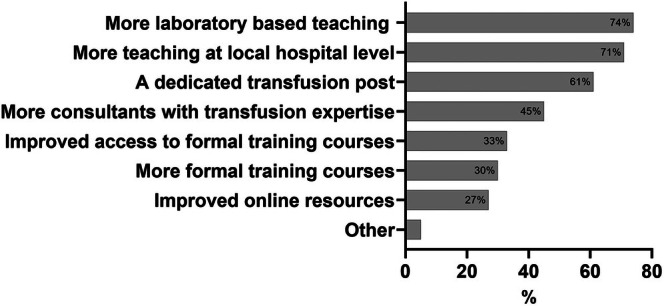
Solutions to improve transfusion training. Solutions to improve training selected by respondents (*N* = 150). ‘Other’: More exposure to transfusion cases (*n* = 2); reduce course fees (*n* = 1); improved blood service course availability pre‐examinations (*n* = 1); national course at the beginning of ST3 (*n* = 1); separate training programme for transfusion (*n* = 1); two separate shorter transfusion posts early and late in training (*n* = 1).

The most frequently suggested solution to improve training was more laboratory‐based teaching, followed by more teaching at a local hospital level, and a dedicated transfusion post (Figure 3). Illustrative free text comments relating to solutions for improvement from three different trainees are listed here.

Solutions free text comment 1:

(The) formal training courses are already excellent and accessible. I've not used other online resources but would consider depending on quality and time. Preservation of local transfusion teaching in hospital and lab is essential for our training. Formal/online courses should be considered adjuncts.

Solutions free text comment 2:

Transfusion training needs to be integrated into the daily practice and we need more resources in our trust.

Solutions free text comment 4:

There should be a dedicated transfusion post in every region. Transfusion is relevant/underpins most haematology disciplines and we should be appropriately trained in this area to reflect this.

## 4 DISCUSSION

The survey results provide a detailed picture of current transfusion training from the perspective of haematology trainees across the UK. It is a call to action to provide consistent and effective transfusion training for all trainees to potentially improve transfusion safety for patients. This is now even more urgent given the IBI recommendations. Transfusion represents a significant part of both the JRCPTB haematology training curriculum and the FRCPath Haematology examinations.^12^, ^13^ Haematology trainees must have appropriate knowledge and skills to inform their own practice, to influence the transfusion practice of many other health care professionals via liaison work and some of whom will be responsible for hospital transfusion service delivery as future consultants.

The last national survey of transfusion training was conducted in 2008, and noted the median length of transfusion posts had decreased from 6 to 3 months over the preceding decade.^6^ In our survey, the most common duration of training posts reported was 1–3 months, so this may represent a further reduction. In comparison to the 2008 results which concluded there was often ‘little or no hospital training’, this survey shows that the majority of trainees are gaining some transfusion exposure in hospital settings. The dedicated transfusion courses provided by the blood services were well regarded in both the 2008 and current survey. There was a smaller snapshot survey published in 2012, of 29 delegates at NHS Blood and Transplant transfusion courses, which highlighted many trainees were relying on these courses to deliver most of their transfusion training, and highlighted inadequacies in gaining clinical practical experience with transfusion.^7^ It is disappointing that the current picture of training more than 10 years after these previous surveys also demonstrates mixed satisfaction with transfusion training.

Less than half of the survey respondents had undertaken a dedicated transfusion post or expected to undertake one. It is difficult to see how many transfusion competencies, which rely on spending time with transfusion clinicians and scientists, can be obtained without dedicated transfusion training posts. Some examples of required transfusion competencies are developing proficiency in managing patients with complex transfusion requirements, less common transfusion reactions, emergency transfusion where the ideal components are not available, adverse event review and laboratory management, and familiarity with the legal and regulatory framework in which transfusion medicine is practised.

It is striking that a lack of transfusion laboratory exposure was the most frequently reported barrier, indicating that laboratory aspects of transfusion training in particular need attention. A newly qualified haematology consultant may be tasked with leading a hospital transfusion laboratory, and in district general hospitals may conceivably have limited senior consultant support with this. Providing out‐of‐hours cover necessitates the consultant on‐call to have the confidence and knowledge in dealing with emergency transfusion queries and being able to support the transfusion laboratory. UK haematologists are trained in both clinical and laboratory aspects of haematology.^4^ However, the specialty of clinical haematology has evolved significantly over recent decades, with more consultants becoming sub‐specialists rather than generalists, and not necessarily having any laboratory sessions.^4^ The time trainees spend in other laboratory‐based training activities, notably microscopy, has been felt to be diminishing over time.^4^ Additionally, in recent years, biomedical scientists working in transfusion laboratories have reported critically low staffing levels and high levels of work stress.^14^, ^15^ Given these pressures, laboratory staff do not have time to consistently facilitate haematology trainees in hospital transfusion laboratories. In addition, from our experience, time‐stretched biomedical scientists are more likely to directly phone a consultant with clinical queries rather than phoning the registrar, which might introduce delays if they need to liaise with the consultant, which is another route by which registrars miss out on training opportunities.

Clashing clinical commitments taking priority over transfusion training was frequently cited as a barrier to transfusion training. Haematology as a specialty is facing a workforce crisis with many consultant vacancies and a previous reduction in trainee numbers, at a time when clinical workloads are increasing due to an ageing population and significant diagnostic and therapeutic advances.^16^ Staffing issues, when unaddressed, can significantly compromise the safety of transfusion practices, leading to preventable errors and jeopardising patient outcomes.

One solution could be to ensure funded places in blood service courses for all trainees, which is currently the case in England and Scotland only. There could also be expansion of other established transfusion training programmes, such as Transfusion Camp, which has been shown to improve transfusion knowledge.^17^ Such programmes, however, rely on consultant time and expertise to deliver this training, both of which are in short supply. E‐learning is a growing area, and could be particularly useful for topics which we have established trainees are getting lower levels of exposure to, including laboratory training, antenatal, and paediatric transfusion.^18^ However, it should be acknowledged that part of the goal of delivering transfusion training is to inspire the next generation of transfusion specialists, and this is unlikely to occur as a result of an e‐learning experience. Additionally, only 27% of our respondents thought improving online resources was a good solution.

There is also a need to ensure practical transfusion exposure with dedicated transfusion posts for all and maximising hospital‐based transfusion opportunities. Of note in this survey, whilst transfusion reaction queries were the most frequent type of query handled, only 80% of senior trainees (ST6/7) had helped investigate a transfusion reaction or event. Encouraging and facilitating trainees to be able to follow up on some of these calls would be a relatively straightforward way of improving hospital transfusion training. Less than half of the trainees reported having attended either a hospital transfusion team or hospital transfusion committee meeting, and most identified this to be a useful educational experience. Promotion of trainees attending these meetings should be another relatively easy way for trainees to gain both direct hospital transfusion experience and introduce them to transfusion team members, which may lead to further training opportunities. As these opportunities are already available, it is puzzling that not all trainees have accessed them. There is recently updated transfusion guidance from the JRCPTB which signposts potential training opportunities within hospitals, about which we do not know how frequently it is used or how well publicised this is, and further promotion of this may be worth doing.^19^

The results of this survey have already been used to secure funding and training numbers for additional dedicated transfusion posts in three training regions. The results have informed content creation for the transfusion section of the RCPath portal and the NHS Blood and Transplant (NHSBT) essential transfusion medicine laboratory medicine workbook.^20^

We restricted this survey to transfusion training only, enabling a detailed picture of this to be established, beyond the limited capacity of the annual General Medical Council (GMC) national training survey^21^ to ask specialty‐specific questions, and our approach could be used for similar focused surveys of training in other haematology sub‐specialties. However, we are aware that the lack of comparator with other haematology training sub‐specialities could conversely be seen as a limitation. Whilst definitions of teaching and training were not provided in our survey, we presumed most respondents would interpret teaching as discrete educational sessions and training as the broader provision of learning opportunities. We recognise that our survey cascade methods mean we may not have reached every eligible trainee and there is higher representation from some training regions than others which may bias the results. Despite this, the survey results validate the concerns raised in the informal feedback previously received.

## 5 CONCLUSION

This survey highlights mixed satisfaction among trainees in the adequacy of transfusion training provided to UK haematology registrars. Laboratory aspects of transfusion in particular are often missed and will need to be covered sufficiently and consistently across the UK. Ensuring remedial measures are taken to ensure essential transfusion training for haematology trainees, which will promote safe patient care and better transfusion decisions. Several tangible actions have been identified from this survey, and some have already been realised, including additional dedicated transfusion posts and tailored educational resources.

## AUTHOR CONTRIBUTIONS

LC led the survey distribution, analysed the responses, and drafted the manuscript. LC, LN, SA, DF, SM, SN developed the survey tool, interpreted the survey findings, and contributed to and approved the final manuscript.

## FUNDING INFORMATION

The authors received no financial support for this study.

## CONFLICT OF INTEREST STATEMENT

The authors declare no financial conflicts of interest.

## Supporting information


**Data S1.** Supporting Information.

**Data S2.** Tables S1–S3.

## Data Availability

The data that support the findings of this study are available from the corresponding author upon reasonable request.
