# Supplementary material for: Transfusion training for haematology registrars: Results of a UK‐wide survey
Source: Transfus Med. 2025 May 19;35(4):330–6. doi: 10.1111/tme.13146 (PMC12361835; doi:10.1111/tme.13146)
Supplement: Supplementary file 1 — Data S1. Supporting Information. [file TME-35-330-s002.docx]

# Supplementary Information File 1: Survey tool

**National Transfusion Training Survey for Haematology SpRs**

This national survey of transfusion training for haematology specialty trainees is on behalf of the RCPath Transfusion Specialty Advisory Committee and has the support of the JRCPTB Haematology Specialist Advisory Committee and the British Society of Haematology Education Committee. The results of the survey will be used to inform future transfusion training.

The survey is anonymous and in order to protect anonymity we will not report

results separately in rotations with less than 3 answers. Please note this survey is only for haematology registrar doctors registered in a specialty training programme.

**1. What stage of training are you?**

If you are a haematology specialty trainee undertaking an out-of-programme activity, then please answer with your stage prior to commencing your out-of-programme period.

ST3

ST4

ST5

ST6

ST7

**2. Have you passed FRCPath?**

Yes, parts 1 and 2

Part 1 only

Neither part yet

**3. In which deanery are you based? If currently out-of-programme, please answer with the deanery in which your national training number is registered.**

Defence Postgraduate

East Midlands

East of England

London

KSS

Mersey

North East

North Western

Northern Ireland

Scotland - North

Scotland - East

Scotland - South East

Scotland - West

Severn

Peninsula

Thames Valley

Wales

Wessex

West Midlands

Yorkshire and Humber

**4. If your region is further subdivided by rotations (eg. London – North Central, East Midlands - North), please specify your rotation**

**5. Have you undertaken a dedicated transfusion post?**

Yes

No

Not yet, but I will in due course

**6. Where was the transfusion post based?**

In a hospital

At the Blood Service

Both

Not applicable, I have not undertaken a dedicated transfusion post

**7. How long did the transfusion post last for?**

1 week or less

More than 1 week to (or equal to) 1 month

More than 1 month to (or equal to) 3 months

More than 3 months to (or equal to) 6 months

More than 6 months

Not applicable, I have not undertaken a dedicated transfusion post

**8. To what extend do you agree with the following statement: ‘My transfusion post was useful educationally’**

Strongly agree

Agree

Neither agree or disagree

Disagree

Strongly disagree

Not applicable, I have not undertaken a dedicated transfusion post

**9. Outside of a dedicated transfusion post, have you received transfusion-focused teaching in the hospitals where you have worked?**

Yes

No

**10. Please state how often the transfusion teaching took place?**

**11. Did the teaching cover laboratory aspects of transfusion?**

Yes

No

**12. Did the teaching involve spending time in the hospital transfusion**

**laboratory?**

Yes

No

**13. Have your regional training days covered transfusion topics?**

Yes

No

**14. Have you dealt with clinical transfusion issues/queries at work?**

Yes, as part of routine clinical work

Yes, while on call

Yes, both in hours and on call

No

**15. Which types of clinical transfusion issues/queries have you been involved in managing at work?** (Tick all that apply)

Transfusion reactions/adverse events

Major haemorrhage/emergency transfusion

Patients with special transfusion requirements/special components

Identification and management of clinically significant red cell antigen and

antibodies

Antenatal transfusion

Paediatric transfusion

Therapeutic apheresis, stem cell collection and exchange transfusion

Alternatives to blood transfusion

Not applicable, I have not managed transfusion queries at work

**16. Have you ever helped investigate a transfusion reaction or adverse**

**event?**

Yes

No

**17. Have you attended Hospital Transfusion Team (HTT) or Hospital Transfusion Committee (HTC) meetings?**

HTT

HTC

HTT and HTC

Neither

**18. If you have attended either and HTT or HTC meeting, how often have you attended?**

**19. If you have attended HTC and/or HTT meeting, to what extent do you agree that it was useful educationally?**

Strongly agree

Agree

Neither agree or disagree

Disagree

Strongly disagree

Not applicable, I have not attended an HTC or HTT meeting

**20. Have you attended a transfusion medicine course run by the Blood Service?**

Yes

Not yet, but I plan to

No, and I do not plan to

**21. Please give details of which courses you have attended** (Tick all that apply)

NHSBT Essential Transfusion Medicine

NHSBT Intermediate Transfusion Medicine

NHSBT RCPath Pre-exam Revision

Other (please comment)

**22. Have you used online transfusion resources or e-modules**

Yes

No

If yes, please give details

**23. Do you feel equipped to handle transfusion issues that arise within normal working hours?**

Yes, fairly independently

No, but I can always ask a consultant for advice

No, and there is a lack of senior support

Unsure

**24. Do you feel equipped to handle transfusion issues that arise while on-call?**

Yes, fairly independently

No, but I can always ask a consultant for advice

No, and there is a lack of senior support

Unsure

**25. Do you think your training programme will equip you to support a hospital transfusion laboratory at consultant level?**

Yes, for clinical and laboratory management aspects

Yes, for clinical aspects only

No

Unsure

**26. Do you think your training programme will equip you as a consultant to provide safe clinical transfusion advice to colleagues in other specialties?**

Yes

No

Unsure

**27. Overall, how satisfied are you with your transfusion training?**

Very satisfied

Satisfied

Neutral

Dissatisfied

Very dissatisfied

**28. What are the barriers to transfusion training?** (Select all that apply)

Lack of provision of dedicated transfusion training

Other clinical commitments take priority over transfusion training

Lack of availability of consultant supervision

Lack of exposure to the transfusion laboratory

Lack of exposure to clinical cases

No barriers

Other (please specify)

**29. What would most improve transfusion training?** (Select all that apply)

More formal training courses

Improved access to formal training courses

More laboratory-based teaching

More teaching at local hospital level

Improved online resources

A dedicated transfusion post

More consultants with transfusion expertise available to deliver training

Other (please specify)

**30. Do you have any other comments about transfusion training?**
