# Supplementary material for: Transfusion training for haematology registrars: Results of a UK‐wide survey
Source: Transfus Med. 2025 May 19;35(4):330–6. doi: 10.1111/tme.13146 (PMC12361835; doi:10.1111/tme.13146)
Supplement: Supplementary file 2 — Data S2. Tables S1–S3. [file TME-35-330-s001.docx]

# Supplementary Information File 2: Additional results

## Supplementary Table 1: Transfusion queries managed

| **Transfusion query category** | **N (%)** |
| --- | --- |
| Transfusion reactions/adverse events | 141/150 (94.0) |
| Patients with special transfusion requirements/special components | 139/150 (92.7) |
| Major haemorrhage/emergency transfusion | 131/150 (87.3) |
| Therapeutic apheresis, stem cell collection and exchange transfusion | 90/150 (60.0) |
| Alternatives to blood transfusion | 94/150 (62.7) |
| Identification and management of clinically significant red cell antigen and antibodies | 89/150 (59.3) |
| Antenatal transfusion | 49/150 (32.7) |
| Paediatric transfusion | 40/150 (26.7) |
| Not applicable, I have not managed transfusion queries at work | 1/150 (0.7) |

## Supplementary Table 2: Transfusion courses attended

| **Transfusion course** | **N (%)** |
| --- | --- |
| NHSBT Essential Transfusion Medicine | 125/150 (83.3) |
| NHSBT Intermediate Transfusion Medicine | 89/150 (59.3) |
| NHSBT RCPath pre-exam revision | 55/150 (36.7) |
| Scottish National Blood Transfusion Service course | 4/150 (26.7) |
| Welsh Blood Service course | 3/150 (2.0%) |
| Other: *“Basic Introduction to Blood Transfusion”* | 1/150 (0.7%) |

## Supplementary Table 3: Online transfusion resources used

| **Online transfusion resource listed by trainees** |
| --- |
| Blood service transfusion courses (eg. NHSBT, SNBS, WBS) |
| Blood Academy |
| Blood Assist app |
| Blood Bank guy |
| Blooducation |
| Bright space |
| British Society for Haematology guidelines |
| E-learning for transfusion and transfusion reactions |
| Haembase |
| Hospital guidelines |
| JPAC |
| LearnHaem |
| MHRA |
| NHSBT guidelines/website |
| Online regional teaching sessions |
| SABRE |
| SHOT |
| Transfusionguidelines.com |
| Transfusion handbook |
| Virtualmicroscopy.co.uk |
| YouTube |
| Unable to recall |
